# Supplementary material for: Clinical EEG slowing induced by electroconvulsive therapy is better described by increased frontal aperiodic activity
Source: Transl Psychiatry. 2023 Nov 16;13:348. doi: 10.1038/s41398-023-02634-9 (PMC10651871; doi:10.1038/s41398-023-02634-9)
Supplement: Supplementary file 1 — Supplementary figure and caption text [file 41398_2023_2634_MOESM1_ESM.docx]

**Supplementary Material**


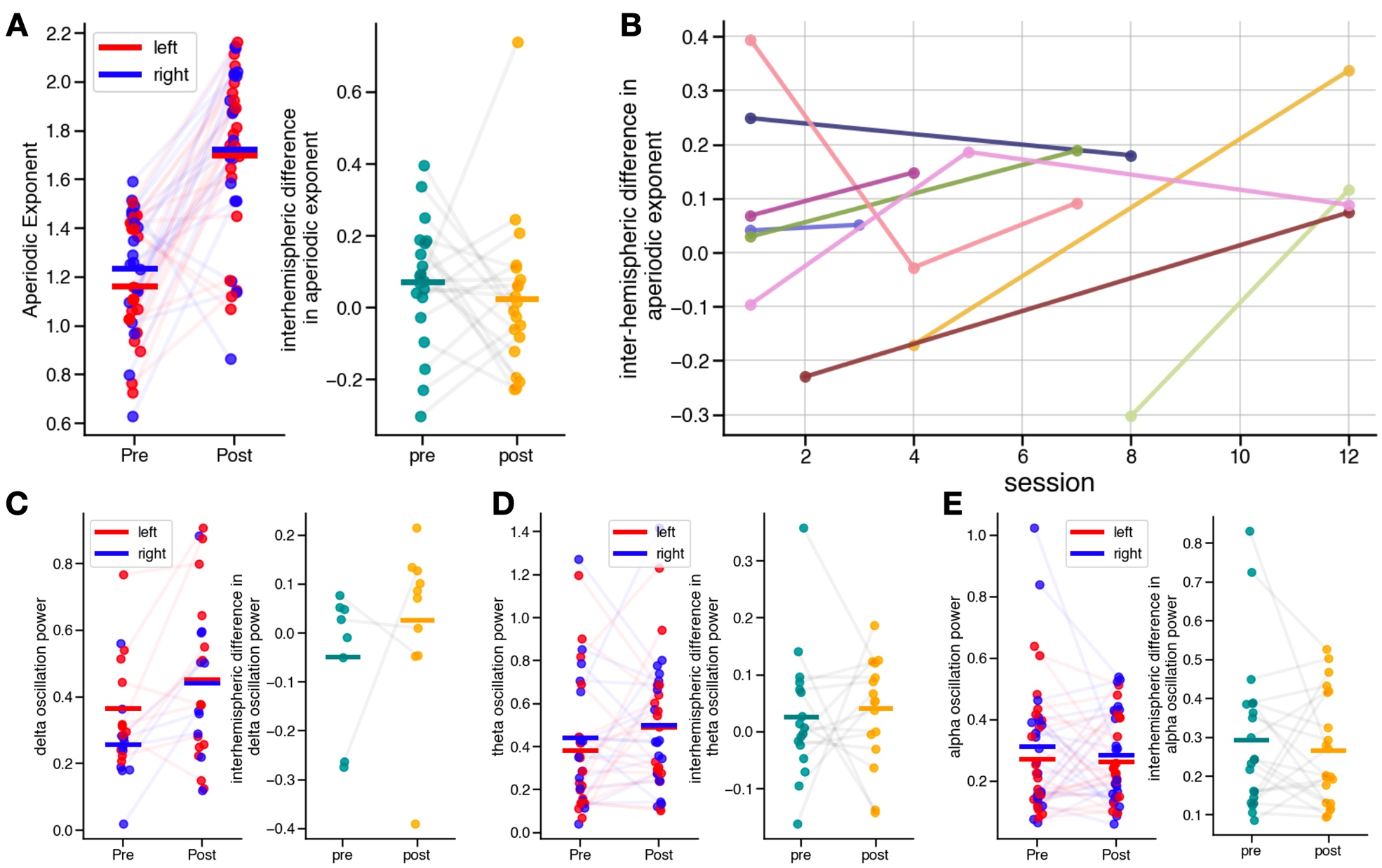


**Fig. 1 | Hemispheric analysis of EEG measures. a,** Acute effects on interhemispheric differences in aperiodic exponent across all patients and sessions, right panel: averaged aperiodic exponent of frontal right and left electrodes pre- and post-ECT, left panel: difference in average aperiodic exponent between right and left hemisphere pre- and post-ECT. **b,** Longitudinal interhemispheric differences within patients throughout treatment. **c-e,** Acute effects on interhemispheric differences in average frontal oscillation peak power in delta (1-3 Hz), theta (3-8 Hz), and alpha (8-12 Hz) ranges. Few patients had an oscillation peak in these ranges for both hemispheres pre- and post-ECT.
